# A more practical guide to incorporating health equity domains in implementation determinant frameworks

**DOI:** 10.1186/s43058-021-00146-5

**Published:** 2021-06-05

**Authors:** Eva N. Woodward, Rajinder Sonia Singh, Phiwinhlanhla Ndebele-Ngwenya, Andrea Melgar Castillo, Kelsey S. Dickson, JoAnn E. Kirchner

**Affiliations:** 1 Center for Mental Healthcare and Outcomes Research, U.S. Department of Veterans Affairs, North Little Rock, AR USA; 2 Department of Psychiatry, University of Arkansas for Medical Sciences, Little Rock, AR USA; 3 South Central Mental Illness Research, Education and Clinical Center, U.S. Department of Veterans Affairs, North Little Rock, AR USA; 4 Philander Smith College, Little Rock, USA; 5 Graduate School, University of Arkansas for Medical Sciences, Little Rock, USA; 6 Department of Child and Family Development, Child and Adolescent Services Research Center, San Diego State University, San Diego, USA; 7 VA Team Based Behavioral Health QUERI, North Little Rock, AR USA

**Keywords:** Health equity, Implementation, Health disparities, Framework, Theory, Implementation science, Determinant

## Abstract

**Background:**

Due to striking disparities in the implementation of healthcare innovations, it is imperative that researchers and practitioners can meaningfully use implementation determinant frameworks to understand why disparities exist in access, receipt, use, quality, or outcomes of healthcare. Our prior work documented and piloted the first published adaptation of an existing implementation determinant framework with health equity domains to create the Health Equity Implementation Framework. We recommended integrating these three health equity domains to existing implementation determinant frameworks: (1) culturally relevant factors of recipients, (2) clinical encounter or patient-provider interaction, and (3) societal context (including but not limited to social determinants of health). This framework was developed for healthcare and clinical practice settings. Some implementation teams have begun using the Health Equity Implementation Framework in their evaluations and asked for more guidance.

**Methods:**

We completed a consensus process with our authorship team to clarify steps to incorporate a health equity lens into an implementation determinant framework.

**Results:**

We describe steps to integrate health equity domains into implementation determinant frameworks for implementation research and practice. For each step, we compiled examples or practical tools to assist implementation researchers and practitioners in applying those steps. For each domain, we compiled definitions with supporting literature, showcased an illustrative example, and suggested sample quantitative and qualitative measures.

**Conclusion:**

Incorporating health equity domains within implementation determinant frameworks may optimize the scientific yield and equity of implementation efforts by assessing and ideally addressing implementation and equity barriers simultaneously. These practical guidance and tools provided can assist implementation researchers and practitioners to concretely capture and understand barriers and facilitators to implementation disparities.

**Supplementary Information:**

The online version contains supplementary material available at 10.1186/s43058-021-00146-5.

Contributions to the literature

- Applications of how the Health Equity Implementation Framework guided other implementation efforts
- Practical tools, including a table of sample measures for health equity determinants, a qualitative interview guide, and a qualitative codebook
- Clarified broad steps for integrating a health equity lens into an implementation determinant framework

## Background

Health equity occurs when all people have socially just opportunities for optimal well-being. Disparities in healthcare implementation exist when a healthcare innovation, such as a program or treatment, is delivered with significantly worse access, receipt, use, quality, or outcomes for certain populations compared to others [1]. Structural factors and systems greatly contribute to different as well as unjust or unfair treatment of certain populations. Populations that experience worse health or healthcare might be defined by race, ethnicity, sexual orientation, gender identity, socioeconomic status, functional limitation, or other characteristics [2]; we refer to these groups as marginalized populations based on social, economic, and/or environmental disadvantage that accompanies health inequities [3]. One example of an implementation disparity in United States (U.S.) pediatric healthcare is screening and diagnosis of autism spectrum disorder. Although there are valid and reliable autism screenings and clear criteria for diagnosis, racial and ethnic minority children who meet the criteria are less likely to be diagnosed than non-Hispanic white children [4]. Thus, effective screenings and diagnoses are implemented inequitably for racial and ethnic minority children, resulting in delayed treatment for children of color. This implementation disparity is exacerbated when children are finally diagnosed properly with autism, as children of color are less likely to receive quality treatment [5]. Unfortunately, several implementation disparities may be undetected. As Braveman wrote, “Health disparities are the metric we use to measure progress toward achieving health equity” [3].

Overall, implementation science has yet to actively and systematically assess, address, and evaluate unique factors contributing to healthcare inequities, including institutional and structural problems, such as racism, that are economic, regulatory, social, historical, and political determinants of implementation for marginalized groups [6]. There are many reasons why implementation researchers have yet to showcase solutions to healthcare inequities including underrepresentation of marginalized and resource-poor communities in implementation studies [6, 7], lack of true engagement with marginalized communities in developing implementation science and practice [8], lack of consistent methods and data elements related to equity across implementation studies [9], and exclusivity and social injustice within the implementation science workforce perpetuated by structures making it harder for institutions to recruit and retain marginalized people (e.g., school-to-prison pipeline). Also, disparities exist for innovations being implemented and, if not adapted for marginalized populations, implementation may perpetuate the exclusion of marginalized communities and widen health inequities [6]. Similar to implementation studies, marginalized populations have historically been excluded from clinical trials and efficacy studies [10]. Further, innovations are often not designed nor as efficacious for marginalized populations [11–13]. Thus, the limitations of disparities in innovation development can be inherited by implementation science and likely perpetuated if the implementation does not systematically consider disparity determinants, cultural adaptations, and other ways to ensure health equity.

Outside the U.S., health equity and implementation research predominantly focus on a specific marginalized population, which is an important and valid path toward equity [9, 14–16]. Examples in low- and middle-income countries include measurement tools normed with participants from those countries [17], adapting innovations or delivery methods specifically to those populations [18] and reviewing or developing frameworks specific to those countries [14, 19, 20]. Although adaptations to local contexts are important, there remain gaps in applying principles of health equity to implementation research broadly, partly because locally adapted frameworks are not easily generalizable to other countries or contexts. The current charges to implementation researchers to ensure health equity in their efforts [6, 21] are not possible without adapting implementation determinant frameworks to first capture and understanding barriers to equitable implementation.

### Implementation determinant frameworks with an equity focus are needed

Implementation science frameworks have been categorized into three types: determinant (establishing what factors determine or predict implementation success), process (clarifying how to address determinants to achieve implementation success), and evaluation (determining metrics and assessment to know when implementation success is achieved) [22]. Implementation determinant frameworks are key to inform study design and selection of strategies to match contextual needs; yet, we have only recently considered determinants unique to health inequities, starting with the Health Equity Implementation Framework [23]. We first piloted health equity domains within the context of a determinant framework as this type of framework represents the key first step to detecting (and eventually addressing) implementation disparities. If implementation researchers and practitioners could meaningfully and practically assess and understand the determinants of implementation disparities, this would allow them to adapt the innovation and implementation strategies for marginalized populations and detect health equity determinants as potential moderators for implementation success/failure [21]. Unfortunately, most implementation determinant frameworks have yet to be explicitly adapted for or tested within health equity efforts and any that do appear too vague to be used meaningfully [24].

Our prior work documented and piloted adaptations of one existing implementation determinant framework with three health equity domains to create the Health Equity Implementation Framework [23]. One may also use the Health Equity Implementation Framework in its entirety as an implementation determinant framework or use the three health equity domains as additions to another implementation determinant framework. Many researchers and practitioners have requested clarification of the Health Equity Implementation Framework domains for practical use. Damschroder argued that implementation frameworks must describe how domains are well-grounded in existing literature, provide clear definitions, and offer suggested validated implementation strategies [22]. Therefore, we review definitions of the domain of the Health Equity Implementation Framework in more depth than in prior work, showcase two applications of this determinant framework from the literature, and delineate steps to incorporate health equity domains in an implementation determinant framework, with sample measures and data collection tools for each domain.

### Health Equity Implementation Framework

**Fig. 1:**
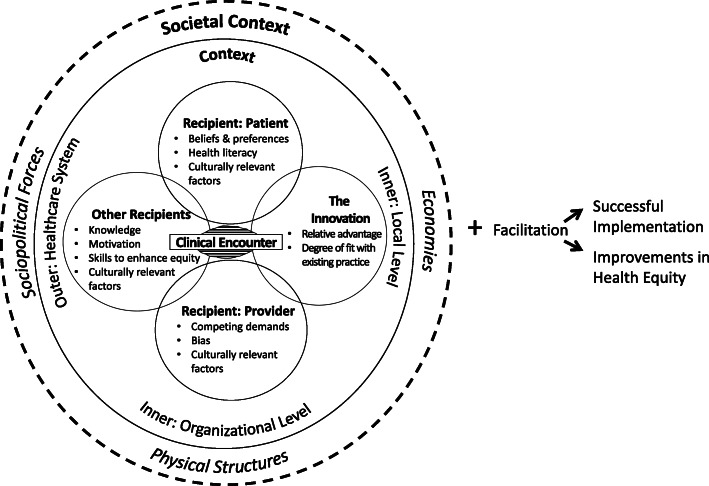
Health Equity Implementation Framework

In the Health Equity Implementation Framework, we proposed *determinants* believed to predict successful *and* equitable implementation, seen in Fig. 1 [23]. These determinants are grouped under *domains*. We define domains as broad constructs relevant to implementation and health equity success. Within each domain are several determinants or specific factors that are measurable and, together in constellation with other determinants, clarify barriers, facilitators, moderators, or mediators to implementation and health equity success. This framework was developed for healthcare and clinical practice settings [25]. In the Health Equity Implementation Framework, we added three health equity domains to the Integrated Promoting Action on Research in Implementation in Health Services (i-PARIHS) framework [26], which also proposes a process—facilitation—by which change in each domain would occur [25, 26]. The focus of this manuscript is on the three health equity domains, rather than facilitation, as science is still emerging on how implementation processes should be tailored or adapted to promote equity.

### Domains typical in implementation determinant frameworks

Broad domains typical in implementation determinant frameworks focus on factors spanning multiple levels, including the individual (e.g., personal characteristics, actors of implementation, individuals receiving an innovation), organization (e.g., clinical service, school, department, factory), community (e.g., local government, neighborhood), system (e.g., school district, hospital system), and policy (e.g., state government, broader laws) [27]. These domains can be further specified, such as inner setting or outer setting within an organization [28]. Domains from i-PARIHS are the basis of the Health Equity Implementation Framework and include those typical in most implementation determinant frameworks [27]. Determinants within each domain act to enable or constrain implementation and each domain is briefly defined below.

#### Innovation

Innovation refers to the treatment, intervention, practice, or new “thing” to be implemented, adopted by providers and staff, and delivered to patients [29]. The innovation may be a program, practice, principle, procedure, product, pill, or policy [30].

#### Recipients

Recipients are individuals who influence implementation and those who are affected by its outcomes, both at the individual and collective team levels [26]. In healthcare, recipients are typically grouped into providers and other staff, and patients and caregivers.

#### Context

Context includes different micro, meso, or macro levels that correspond to inner and outer contexts [26]. Context can include factors such as resources, culture, leadership, and orientation to evaluation and learning. In this framework, the micro-level includes the local inner context (e.g., specific ward or clinic), whereas the meso includes the organization (e.g., hospital or medical center). The macro-level of outer context includes the wider healthcare system and effect this has on the other domains (e.g., United Kingdom National Health Service) [28].

#### Facilitation or process

There are processes by which barriers in implementation domains are solved or overcome, and strengths are harnessed to promote the use of an innovation in routine practice [28]. In i-PARIHS, facilitation is the “active ingredient” or process [31]. Facilitation involves implementation strategies that result in implementation coming to fruition [32, 33].

#### Domains known to affect health equity

The Health Equity Implementation Framework incorporates these domains known to affect health disparities and thus, equity: (1) culturally relevant factors, such as medical mistrust, demographics, or biases of recipients [34–37]; (2) clinical encounter or patient-provider interaction [38–40]; and (3) societal context including physical structures, economies, and social and political forces [41–43]. We added these three health equity domains, described below, from existing research that have clear, strong associations with disparities in health status, access to, quality of, or outcomes of healthcare, [44] or there is enough evidence to suggest determinants within these domains should be considered (e.g., [45]).

1. Culturally relevant factors of recipients. Recipients in the implementation process are individuals who will be asked to offer or receive an innovation (e.g., patients, providers) [26]. Culturally relevant factors of recipients are characteristics unique to a group of people in the implementation effort (e.g., patients, staff, providers) based on their lived experience. Some examples of recipient factors that may be culturally relevant are implicit bias, socioeconomic status, race and/or ethnicity, immigrant acculturation, language, health literacy, health beliefs, or trust in the clinical staff or patient group [36, 37]. Demographic characteristics, such as socioeconomic status or race, are not inherently descriptive of one’s culture. Rather, the important takeaway is how living in the world with these factors shapes one’s culture and experience (e.g., living in impoverished neighborhoods, experiencing racism). We do not feel strongly that any demographic factor be categorized as a culturally relevant factor—the most important thing, from our view, is that implementation practitioners and scientists acknowledge these demographics among their recipient groups and consider how implementation may need to be adapted based on the lived experience of recipient groups. For instance, implementation practitioners and scientists should consider how implementation would need to change for those who have little formal education, are people of color, or are underinsured. Factors from patients and providers might attend to differences between, for example, age, pre-existing stereotypes, or lack of trust that could hinder the interaction [40]. Culturally relevant factors will vary by group, local context, and individuals. It is crucial that culturally relevant factors of recipients are considered as determinants or potential moderators in implementation success/failure when patients belong to a group experiencing a health or healthcare disparity.
2. Clinical encounter (patient-provider interaction). This domain describes the transaction that occurs between patients and providers in healthcare appointments, where decisions concerning diagnoses and treatment are made, and providers administer care [46]. The clinical encounter is important to assess because there is a myriad of behaviors and perceptions during the clinical encounter that affect whether an innovation is offered by a provider and whether it is accepted by a patient. Behaviors will vary by innovation, context, and recipients and may be especially important for patients who experience health or healthcare disparities due to unequal power between them and providers. Factors to measure might be how recipients maneuver the conversation accordingly to achieve their individual and shared goals [40, 47]. It would also be important to capture unconscious or implicit bias from either recipient about the other recipient’s characteristics, such as race, weight, or perceived sexual orientation [48–50]. These unconscious biases may manifest in unhelpful behaviors during the encounter, such as dismissing someone’s concerns, interrupting the other person, or not smiling, touching, or making eye contact. Clinical encounters predict patient satisfaction, trust, and health outcomes; thus, it is crucial to assess and address what occurs during the clinical encounter, especially with regard to implementation disparities [47, 50–52].
3. Societal context: economies, physical structures, and sociopolitical forces. This domain is similar to social determinants of health, yet also incorporates more upstream determinants (e.g., governance) that have been investigated less relative to mid- or downstream determinants (e.g., neighborhoods) [44]. Societal context includes three specific determinants: (1) economics, (2) physical structures, and (3) sociopolitical forces. In piloting the Health Equity Implementation Framework, societal context affected receipt of antiviral hepatitis C virus medicine for Black patients in the U.S. Veterans Health Administration [23].

Societal context may include historical or current discrimination against marginalized groups, such as racism, classism, or transphobia that may be formally or informally institutionalized within any organizational or local context. These factors usually occur in the broadest levels of the environment (e.g., province, nation), affecting the healthcare system, clinics, and recipients downstream. Many societal context determinants are interrelated, such as a policy affecting a physical structure. It is not as important to distinguish whether a factor is exclusively an economy, physical structure, social norm, or all three; rather, it is important these societal determinants are detected and addressed to ensure strategies address key drivers of societal inequities. Societal context may not be assessed comprehensively in one study or initiative, due to feasibility constraints, but they should be documented in formative evaluations/initial diagnostic assessments of the implementation problem.

#### Economies

There are four typical structures of economies including a traditional economy (i.e., mostly agricultural), market economy (i.e., firms and private interests control capital), command economy (i.e., government controls capital), and a mixed economy (combination of command and market) [53]. It is helpful to consider how economic structure affects access to resources for implementation. Market forces can be used to change demand for products deemed healthy or unhealthy, therefore driving policy implementation. Examples of market forces include taxes on tobacco, unhealthy food, and soft drinks, or food subsidy programs for women with low incomes [41].

#### Physical structures

Equity can be affected by how physical spaces, or “built environments,” are arranged and how transition between those spaces occurs for healthcare [41]. Physical structures include any factors where people have to physically go to get healthcare and what environmental elements people may be exposed to (e.g., privacy or lack thereof, what they see, what is emitted in the air and into their bodies). One example in healthcare settings is the type and quality of language translations of information displayed (e.g., flyers, waiting rooms)—whether it matches the language of patients served [54]. The location of the healthcare setting in a town or city is important in relation to where patients reside [54, 55], e.g., is it difficult for patients to get to the point of care? Another example is the implementation of one U.S. state’s naloxone standing order in which pharmacies could distribute naloxone without a prescription: 61.7% of retail pharmacies had naloxone available without a prescription [56]. However, naloxone availability was lower in neighborhoods with higher percentages of residents with public health insurance—a physical structure problem (lower availability of naloxone in some neighborhoods) interacting with an economic factor (public health insurance). This finding was particularly problematic due to an increased cost of naloxone for people on public health insurance as a result of the statewide mandate.

#### Sociopolitical forces

The third societal context describes social norms or political forces, which can include but are not limited to political support, laws, and social structures in which linkages between institutions perpetuate oppression, such as racism, misogyny, classism, or heterosexism [43, 57]. For instance, public health policies (e.g., fiscal, regulation, education, preventative treatment, and screening) demonstrate positive and negative effects on health disparities that occur across health domains (e.g., tobacco, food and nutrition, reproductive health services) [41]. As another example, a study examined U.S. state legislators’ behavioral health research-seeking practices and dissemination preferences and found significant variation between Democrats and Republicans, suggesting dissemination materials be tailored to different social norms for different groups [58].

Next, we showcase two examples of how implementation teams have used culturally relevant factors of recipients, patient-provider clinical encounter, and societal context as health equity domains in formative and process evaluations. Each example comes from different health service sectors and describes efforts focused on implementation disparities.

### Conducting a formative needs assessment prior to implementation

The Health Equity Implementation Framework has been applied to guide a needs assessment for an implementation project aiming to reduce inequities in the provision and receipt of publicly funded services for individuals with developmental disabilities in the U.S. (Rieth SR, Dickson KS, Plotkin R, Corsello C, Ko J, Cook-Clark T, et al: An in-depth analysis of expenditures for Latinx individuals with developmental disabilities: Following the money and perspectives from the front line, in preparation). In 2016, the State of California Department of Developmental Services made funds available to address significant inequities in service expenditures for Latinx clients. In response, the San Diego Regional Center, the local agency coordinating and funding publicly-funded developmental disability services, initiated a partnership with local services and implementation researchers to identify inequity reduction targets and develop and implement an inequity reduction model. A mixed methods needs assessment was conducted to inform model development and implementation activities. Quantitative data included administrative data from the previous year. Qualitative data were gathered from focus groups with regional center case managers to identify key determinants of inequities from their perspectives.

The Health Equity Implementation Framework guided the identification of implementation determinants and the selection of data coding and analyses. Specifically, the framework informed the development of the qualitative codebook, including coding domains and definitions that were iteratively refined for this project. The framework guided subsequent integration of qualitative and quantitative data, including the use of qualitative themes to complement and expand quantitative findings. Preliminary findings indicate a significant impact of outer and inner context on inequities, including fit between patient recipient characteristics, culturally relevant factors, and characteristics of available innovations. Additional outer context factors, including sociopolitical factors and physical structures such as location (urban versus rural) also impact service utilization, including interactions with provider factors and innovation characteristics.

### Conducting a process evaluation to categorize ongoing barriers/facilitators

In Toronto, Canada, legally sanctioned supervised consumption services (the innovation) are integrated within health centers; implementation has occurred and is ongoing. Supervised consumption services are for people who inject drugs to receive sterile injection equipment and inject under staff supervision. Staff educate on safer injecting, provide referrals to services, and can respond to overdoses, reducing transmission of infectious diseases (e.g., HIV) and overdose deaths. Researchers used ethnographic observation and individual semi-structured interviews with 24 patients who injected drugs in supervised consumption services at two community health centers, half of who were people of color or Indigenous to Canada [59]. After coding, researchers interpreted findings within domains of the Health Equity Implementation Framework.

Integrating legally sanctioned supervised consumption services within health centers (sociopolitical force) provided clients access to other health services, including dentistry and medical assistance that eliminated the need for a provider visit (characteristics of the innovation, organizational context). Patients appreciated having everything in one physical place (physical structure). One participant said the services allowed them to avoid meeting providers who were prejudice against drug use (sociopolitical force, provider culturally relevant factor).

Yet, there were barriers to implementation. Patients were uncomfortable being seen by peers using the center due to stigma about drug use (sociopolitical force). Spatial limitations at the center made it difficult to have privacy while injecting (physical structure). Patients preferred the center to be open all the time (organizational context), but there were not enough staff for that flexibility (healthcare system context). Ethnographic observation suggested standalone supervised consumption services were consistently busier than integrated services, potentially because some people felt uncomfortable in a healthcare setting (patient factor).

## Methods

We completed a consensus process to clarify steps for incorporating a health equity lens into an implementation determinant framework, situated within the existing literature. We reviewed Moullin and colleagues’ ten suggested steps for incorporating frameworks into an implementation effort [60] and selected the five steps applicable to an implementation determinant framework (vs. evaluation or process frameworks). The first author (ENW) expanded those five steps from Moullin and colleagues [60] with steps on how to incorporate health equity domains and determinants. These steps were vetted with the authorship team through a process of oral discussions, reviewing written documents, and refining steps until all agreed. Next, our team created or aligned a table, tool, or example for more practical guidance on how each step could be executed.

## Results

### Applying health equity domains across an implementation effort

Below are suggested steps on how to use frameworks in an implementation effort [60] with a focus from our authorship team specifically on health equity in an implementation determinant framework.

#### Select a suitable framework or domains for an implementation disparity problem

If an implementation effort will focus on a health condition or marginalized population with documented health or healthcare disparities, we strongly suggest incorporating determinants from the three health equity domains into one’s preferred implementation framework or use the Health Equity Implementation Framework. If we do not assess or consider domains that promote or inhibit disparities, then we cannot expect to address them in a meaningful way, and we cannot build our scientific integration of health equity and implementation science to generalize across implementation efforts. To find an implementation determinant framework other than the Health Equity Implementation Framework that can be adapted for implementation disparities, pick a framework using an online webtool showcasing many implementation determinant frameworks (https://dissemination-implementation.org/) [61].

The Health Equity Implementation Framework can be adapted to any population or country where implementation disparities occur. The framework proposes determinants of inequitable implementation and a process (facilitation) by which to address determinants. The framework has not been used as a process or evaluation framework; thus, we cannot speak to the value of focusing on these domains in implementation processes or to these domains as evaluation outcomes.

#### Determine implementation determinants

Assess which determinants are present in an implementation disparity and whether each determinant is a barrier (challenge) to improving equitable implementation or a facilitator (strength). Through formative evaluation to assess barriers and facilitators in each domain [62, 63] align qualitative interview guides, quantitative measures, and other assessment methods (e.g., participant observation, policy review) to the framework’s determinants. For qualitative and quantitative assessments of determinants, we present in Table 1 a variety of assessment methods and measures one might use to assess determinants within the Health Equity Implementation Framework. An illustrative example is given to showcase how others have assessed various determinants incorporated in the framework. Although Table 1 is not exhaustive, it is a robust reference and guide to consider certain measures, tools, or data sources for formative evaluation.

**Table 1:** Definitions, illustrative examples, and sample measures of the Health Equity Implementation Framework

Domain and determinants	Definition	Illustrative example(s)	Sample measures^a^
**Characteristics of the innovation** [31]: • Underlying knowledge sources • Clarity • Degree of fit with existing practice/values • Usability • Relative advantage • Trialability • Observable results • Evidence for the innovation [64] • Research • Clinical experiences • Patient experiences	An innovation is a treatment, intervention, or practice with unique characteristics that determine how such innovations will be applied in a particular setting. Innovations fall into one of the “7 Ps”: programs, practices, principles, procedures, products, pills, or policies [30]. The innovation should be tailored with minor changes or adapted with major changes to the setting’s needs and practices for successful implementation [31, 65].	A study examined the uptake of the Healthy Heart Kit (innovation), a risk management and patient education resource for the prevention of cardiovascular disease, in a primary care setting. They found that relative advantage (innovation was the most comprehensive tool for cardiovascular health) and observable results (evidence-based practice supports innovation) were more influential to the uptake of Healthy Heart Kit than other characteristics [66].	Quantitative: • Decision-Maker Information Needs and Preferences Survey • Electronic Health Record Nurse Satisfaction Survey [67] • Reports assessing the current status of implementing the innovation, completed by one clinic point of contact or champion [68] Qualitative: • Barriers and facilitators assessment instrument • General practitioners’ perceptions of the route of evidence-based medicine • Knowledge, attitudes, and expectations of web-assisted tobacco interventions [67]
*Clinical encounter (patient-provider interaction)	This is the nature of the interaction between patient and provider. This domain is centered on how the patient and provider choose, adapt, and coordinate the conversation to achieve their shared and personal goals concerning health-related matters [40]. The interaction could be influenced by: • Predisposition features which are individual differences that influence communication that may be objective (e.g., age) and subjective (e.g., self-concept). • Cognitive/affective influences that show how communication is related to strategy (e.g., goals), attributions (e.g., stereotypical), and trust. • Communication influences refer to how the patient and the provider tailor their responses to create a coherent and effective exchange [40].	In studying recordings of HIV patient-provider encounters, there was less psychosocial talk in patient-provider encounters with Hispanic compared to non-Hispanic white patients [39]. In a study on predictors and consequences of negative patient-provider interactions among a sample of African American sexual minority women, authors found racial discrimination was most frequently mentioned, and gender and sexual orientation discrimination were also related to negative patient experiences [50].	Quantitative: • Patient and provider questionnaires about relevant demographics to assess concordance/match between patient and provider • Patient rating about the encounter: Interpersonal Processes of Care Survey [39] • Experiences of Discrimination Scale [69] Qualitative: • Patient qualitative interviews about their experience of care [70, 71] Clinical encounters coded using audiotapes, analyzed using the Roter Interaction Analysis System [39]
**Recipients** [31]: • Motivations • Values and beliefs • Goals • Skills • Knowledge • Time, resources, support • Local opinion leaders • Collaboration/ teamwork • Existing networks • Learning environment • Power and authority • Presence of boundaries	Recipients are individuals who influence implementation processes and those who are affected by implementation outcomes, both at the individual and collective team levels. Recipients can facilitate uptake of an innovation or resist its implementation [31].	See below	See below
*Recipients: providers and staff: Culturally relevant factors include [35]: • Demographics (e.g., neighborhood immigrant status) • Unconscious/implicit bias • Knowledge and attitudes • Skillsets	In a healthcare setting, providers and staff are the people who administer the innovation. A providers’ objectives and beliefs about a patient affect how they behave during the patient-provider interaction [72]. Providers, especially in busy healthcare settings, may be vulnerable to subconscious bias and stereotypes [73].	Physicians who consider themselves “liberal” spent more time giving more information to patients than those who consider themselves “conservative” [40]. Providers may engage in more detailed conversations about the health status of educated patients, yet provide basic explanations for less-educated patients [40]. During a post-angiogram encounter, physicians perceived patients of lower socioeconomic status as having more negative personality characteristics that include lack of self-control and more negative behavioral tendencies [38].	Quantitative: • Implicit Association Test to assess implicit bias [48] • Surveys of relevant practice, knowledge, attitudes, or skills [74, 75] • Colorblind Racism Scale [76] Qualitative: • Analysis of taped conversation between provider and patient [39, 48] • Participant observation [77] • One-on-one interviews [78]
*Recipients: patients: Culturally relevant factors include [34, 35, 45, 79–81]: • Medical mistrust • Health literacy and numeracy • Demographics (e.g., neighborhood, immigrant status) • Socioeconomic status, including household income, net wealth, health insurance status, education level • Expectations about therapeutic relationships • Beliefs and preferences	In a healthcare setting, patients are the people (individuals, families, caregivers) who will actually receive the innovation. Culturally relevant factors are associated with health and healthcare disparities and can include demographic factors, beliefs, information, and biological or genetic conditions related to equitable implementation.	Asian American patients in Hawaii participated less in their medical visits than mainland Americans [82]. Patients with more formal educations are more expressive and tend to want to play a role in the decision-making process than less educated patients [40]. Many patients are unsure about their role in the encounter and the appropriateness of their participation [83].	Quantitative [34]: • Telephone survey of a random sample of residents • Medical Mistrust Index [84] • Measures of underutilization of health services • Health literacy question [85] • Health numeracy question [86] • Appropriated Racial Oppression Scale [87] Qualitative: • Interview about expectations for treatment or the patient-provider-interaction [39, 88] • Interviews about experience seeking care [89]
**Inner context (local)** [26]: • Formal and informal leadership support • Culture • Previous experience of innovation or change • Change mechanisms for embedding innovation • Evaluation and feedback processes	The immediate local setting of implementation. Examples include: • Ward • Unit • Clinic • Hospital department	Among 303 providers working in 49 publicly funded health programs for youths, providers’ perception of certain leadership styles was associated with stronger provider willingness to adopt evidence-based treatments [90]. Pisando Fuerte is a fall prevention program linguistically and culturally tailored for Latino individuals at risk for falls. It is adapted from “Stepping On,” an evidence-based fall prevention program. Fidelity to Pisando Fuerte was subpar; when comparing fidelity between the two sites, fidelity was lower in the site that did not give additional time to implement the program (poor leadership support) and had no experience in organizing programs like Pisando Fuerte (no previous experience of innovation) [91].	Quantitative: • Perceptions of Supervisory Support Scale [92] • Organizational commitment [93] • Readiness for Organizational Change measure [94] • Validated inner setting measures [95] Qualitative [96]: • Site visit • Key informant interviews about inclusivity • Stakeholder meetings or focus groups with providers about their understanding of equitable care • Public forums and listening sessions • Provider and staff interviews to determine actual practice and processes [97]
**Inner context (organizational)** [26]: • Organizational priorities • Senior leadership and management support • Culture • Structure and systems • History of innovation and change • Absorptive capacity • Learning networks	The organizational atmosphere in which the unit or team is embedded.	Hospitals’ adoption of the Culturally and Linguistically Appropriate Services standards focused on retaining translators and adapting culturally and linguistically appropriate materials. However, this adoption did not often include engagement in broader organizational change [98]. Researchers studied a disparity-reduction program in Israel across 26 clinics and 109 clinical teams. After 3 years, they found different inner context configurations of factors predicting disparity reduction. One example of a successful configuration was clinics with a large disparity gap to minimize, high clinic density, high perceived team effectiveness, and focused efforts on tailoring services to their enrollee patients [99].	Quantitative: • Measures of organizational readiness for change [100] • Cultural Competency Assessment Tool for Hospitals [98] Qualitative [101]: • Key informant interviews assessing knowledge/action of policies about equity • Key informant interviews assessing beliefs organization holds about marginalized people • Stakeholder meetings about the importance of equitable care • Public forums and listening sessions [102] • Focus groups
**Outer context (healthcare system)** [26]: • Policy drivers and priorities • Incentives and mandates • Regulatory frameworks or external accreditation systems • Inter-organizational networks and relationships	This is the broader context defined in terms of resources, culture, leadership, and orientation to evaluation and learning. There is an increasing amount of research that shows that inequities in obtaining preventative care among racial and ethnic minorities compared with non-Hispanic whites are due to “organizational characteristics, including location, resources, and complexity of a clinic or practice” [35].	Researchers examined predisposing, enabling, and need factors as predictors of changes in healthcare utilization and found that patients’ experiences differed by group within the healthcare system and impacted their beliefs and attitudes about receiving healthcare, ultimately affecting the extent to which healthcare services were utilized [50].	Qualitative: • Archival analysis, reading and documenting policies, program manuals, or procedural protocols [103, 104] • Interviews with leadership [99] Quantitative: • 15 core measures of healthcare qualit y[105] • Population surveys • Social network analysis of relationships between relevant leadership and/or teams [99] • Existing reports hospital-wide scores on assessments of care and equity, e.g., National Quality Forum or Healthcare Equality Index [106]
*Societal context [41, 42]: • Economies • Physical structures • Sociopolitical forces • Up-, mid-, or downstream social determinants of health [44]	Forces outside the healthcare system that influence all other domains and determinants of implementation may include but be broader than social determinants of health, may focus on the presence of stigma and discrimination such as racism, classism, or transphobia (as examples) and the institutionalization of such discrimination in every determinant of implementation.^b,c^	See below	See below
*Economies [53]: • Traditional • Command • Market • Mixed	The structure of the city, state, or country related to the wealth and resources of people and what is exchanged for healthcare delivery (e.g., insurance). This can be divided into human resources (i.e., labor, management) and non-human resources (i.e., land, capital goods, financial resources, and technology) [55].	In a study assessing longitudinal effects of health insurance and poverty, researchers reported low-income, middle-aged adults in the U.S. with no insurance, unstable coverage, or changes in insurance have higher out-of-pocket expenditures and financial burdens than public insurance holders [107]. In a case study, the presence of chronic kidney disease indicators in the pay-for-performance system in primary care created an incentive for improvement [26].	Quantitative: • Insurance claims data • Gross domestic product [108] • Gross national product [109] • Minimum wage [110] • Population and total employment [111] • Annual average wage level of the primary, secondary, and tertiary industries [112] • Tax revenue as a percentage of total revenue [113] • Interest rate on saving deposits and inflation rate [114] Qualitative [115]: • Key informant interviews about goods and services exchanged [116] • Analysis of comparative economic structure [115]
*Physical structures: • Location • Availability of public transportation • Actual environment of the point-to-care • Language spoken and/or signage • Available structures in one’s neighborhood to use innovation • Grocery stores • Healthcare facilities • Local businesses • Physical infrastructure	The physical environment, structure, location of services, and recipients, also known as the built environment as it relates to equitable implementation [55].	One study compared Black and White Americans who were exposed to the same set of socioeconomic, social, and environmental conditions in an area of one U.S. city. Although there is robust research documenting disparities in hypertension, diabetes, obesity, and use of health services by race among national samples, within the racially integrated city in the study, disparities in these health conditions were either absent or significantly smaller. Thus, the place where people lived had an impact on their health conditions, beyond race [117]. In a qualitative study of transgender individuals’ experiences in residential addiction treatment, researchers observed that residential facilities that split the milieu and housing based on the gender binary may be stigmatizing people who identify as transgender or gender non-conforming [118].	Quantitative: • Indices of segregation [119]^b^ • Public data such as hospitals per capita, public transportation trips per capita, car ownership, revenue dedicated to parks and recreation, transportation, other infrastructure needs, and grocery stores per capita • Center on Budget and Policy Priorities data • State Departments of Finance and Administration [55] Qualitative: • Windshield and walking surveys include assessing infrastructure; surveyors are on foot and take note of the neighborhood related to the physical or built environment [120].
*Sociopolitical forces [41, 43, 57]: • Policy climate • Political support • Laws • Local culture • Social movements or structures such as racism, classism, heterosexism, transphobia^c^	Policies and procedures, formal or informal, in national and local governments that systemically inhibit or promote equitable health.	In a U.S. study on the adoption of behavioral health evidence-based treatment by states, the following were some factors that played a role: state characteristics, state fiscal supports to promote innovation adoption, and state policy that supports to promote evidence-based treatment adoption [57].	Quantitative: • Select measures of determinants of policy implementation, such as visibility of policy actors or policy implementation climate [121] • The State-Level Racism Index [122]^b^ Qualitative: • INCLENS equity lens: examines whether clinical guidelines address health needs and inequities experienced by marginalized groups [123] • Interview questions with recipients about laws, policies, or social movements relevant to the innovation • Archival analysis of policy documents [103, 104]

*Health equity domains adapted to i-PARIHS

^a^Measures or data collection methods are examples from literature; for a repository of implementation science measures, see the Society for Implementation Research Collaboration’s Instrument Review Project [124]

^b^For a repository of measures specific to racism, see Appendix B of Racism: Science & Tools for the Public Health Professional [125]

^c^Implementation scientists should review existing measurement tools specific to health disparities in your area of interest or study to further integrate health equity into implementation

If one is using qualitative methods to determine some or all of the equitable implementation determinants, we provide examples of questions from qualitative interview guides we piloted that are aligned to domains of the Health Equity Implementation Framework (see Additional file 1). If this approach is used, the framework domains are then helpful for designing qualitative codebooks or templates for analysis. We provide a codebook for analysis we piloted that is aligned to the three health equity domains (see Additional file 2). The codebook for the health equity domains can be combined with codebooks of other determinant frameworks, such as Consolidated Framework for Implementation Research [126].

#### Use domains to develop an implementation mechanistic process model or logic model

Determinants in the Health Equity Implementation Framework may directly influence the success and equity of an implementation effort or they may indirectly affect outcomes as mechanisms through which success or equity are enhanced. Using the three health equity domains added to an implementation determinant framework, one may develop theoretically driven hypotheses about which domains, or determinants within them, must change to lead to improved equity and implementation success [127]. These determinants are mechanisms. When working on an implementation disparity problem, this will ensure some mechanisms related to equity *and* implementation are investigated.

To understand the concept of mechanisms of implementation disparities, we consider a hypothetical example of an implementation disparity at one hospital where an evidence-based innovation is received mostly by White people with moderate or high incomes. In this example, the implementation disparity between patients of different races and incomes may be due to (1) the innovation was developed and tested in samples of mostly White people such that it is not acceptable to or effective for Black people (characteristic of the innovation), (2) providers do not offer the innovation as often to Black patients as they do to White patients (clinical encounter), (3) there may not be many Black or lower-income people served at the hospital (outer context), or (4) the hospital is not readily accessible via public transportation to people with lower incomes who do not have motor vehicles (physical structure). There may be some known or unknown determinant within any domain of the Health Equity Implementation Framework contributing to implementation disparities; perhaps providers have unconscious biases toward Black people (a factor within the cultural recipients domain) that lead to them offering the innovation less frequently to Black patients than to White (clinical encounter). The key factor to change would be unconscious bias to affect provider behavior and alter the clinical encounter. To the extent possible, one can hypothesize which factor is the lever for more equitable implementation—which of these factors, if changed, would result in the innovation being received by more people with lower incomes and more Black people at that hospital? These levers are mechanisms of implementation disparities (areas to change with implementation strategies) for more just and equitable delivery of healthcare. Consider these health equity determinants in developing a logic model to explain the implementation process, including its mechanisms of change.

#### Use framework determinants to conduct and tailor implementation

After formative evaluation or initial diagnosis of the implementation disparity is complete, the areas for change will become clear and implementation strategies will need to be selected and tailored to local context and recipients with careful attention to equity and justice. There are many existing ways to use information from formative evaluation to select and tailor implementation strategies [128–130]. To address implementation disparities, explicitly include determinants of inequity in selecting and tailoring strategies, as well as unique barriers that may prevent organizations from addressing these inequities. For example, there may be a need to use community- or patient-informed strategies to repair harm and build trust among patient recipients who have been and are marginalized in healthcare systems, improve cultural and structural competence at all levels of an organization, or continuously monitor reach between patient subgroups to detect change in disparities. Although some are focusing on equity more in using implementation strategies [33, 91, 99, 131], there is considerably more work to be done on this, and careful attention to equity elements is needed to tailor implementation.

As implementation progresses, an implementation plan will need to be adapted as determinants change. The Health Equity Implementation Framework can be useful for determining areas to assess repeatedly and thus, intervene on, throughout implementation. Doing so ensures an equity lens is applied throughout implementation and that implementation processes, such as planning, strategy use, and goal setting, are thoughtfully executed according to dynamic needs. Repeated assessments can be done informally through observations, consultations with recipients and leadership, or more formally through mixed methods, including ones mentioned in Table 1 and used previously in formative evaluation [63].

#### Writing implementation reports or findings

For documenting the results of an implementation effort, clarify how the Health Equity Implementation Framework or its three health equity domains were incorporated. For example, barriers and facilitators from formative evaluation may be presented by framework domains. As implementation progresses, a team may want to document key changes within domains from the Health Equity Implementation Framework, similar to how ongoing implementation barriers and facilitators were recorded for the study that examined the implementation of legally sanctioned supervised consumption services in Canada [59]. The mixed method approaches suggested earlier will provide key information to be reported, making clear why implementation was successful or not, and how certain strategies affected whether disparities in receipt, use, access to, or quality of an innovation were reduced [6].

## Discussion

Disparities in healthcare occur in implementation outcomes and patient health outcomes. Implementation disparities are rooted in social injustice, exacerbated by multiple inputs, such as societal context, patient mistrust, provider bias, and poor patient-provider interactions. The three health equity domains presented in more depth here are key adaptations for implementation researchers and suggested to adapt one’s preferred implementation framework (e.g., EPIS) to incorporate an equity lens and account for inputs contributing to implementation disparities. Three health equity domains from the Health Equity Implementation Framework can be studied as determinants of implementation, as showcased in the application to services for developmental disabilities in California. We propose that an increased focus on health equity explicitly at multiple ecological levels in implementation science and practice will elucidate drivers of health inequities such as structural racism, heterosexism, and patriarchy. Thus, the discovery of these drivers of health inequities should necessitate implementation strategies to overcome or resolve such complex and oppressive structures. Future research should focus on implementation strategies (or other processes) used to address health equity determinants of unjust health inequities in our healthcare systems and societies.

We have only piloted the three health equity domains within the context of a determinant framework; however, they may be suitable as process or evaluation variables. As this framework evolves through implementation research and we have more data to inform its application, future considerations could include that some of these domains for determinants should also be outcomes of implementation disparity reduction efforts. For an implementation process framework that incorporates an equity lens, see frameworks proposed by Nápoles and Stewart [132] and Eslava-Schmalbach and colleagues [133]. For an implementation evaluation framework that incorporates an equity lens, see preliminary equity-focused implementation outcomes [133] and the proposed extension of the RE-AIM framework [134].

There are limitations to our framework and practical guidance presented here. We have piloted test many, but not all, the feasibility and acceptability of the steps we described using three health equity domains and measures in Table 1. However, we suggest these as starting places, and with confidence, as they all have entire bodies of science showcasing their relevance to health equity. We limited the application of this framework to healthcare settings, although it could be adapted to community or school settings. Although health equity can be incorporated across several determinant frameworks, we provided a detailed application of health equity domains tied to i-PARIHS. They have the potential for broader applications to other implementation science frameworks. This has not been piloted yet to our knowledge.

## Conclusion

Implementation researchers and practitioners must adopt a health equity lens as foundational to any research-practice gap where inequity exists. Researchers might collect data on the feasibility, acceptability, and predictive utility of health equity determinants in this burgeoning area of implementation science. The Health Equity Implementation Framework is an implementation determinant frameworks to capture and understand barriers and facilitators to health inequities [23, 135]. The applications, steps, and tools in the manuscript are one step toward systematic integration of health equity and implementation science in frameworks.

## Supplementary Information

**Additional file 1:** Health Equity Implementation Framework Interview Guide: Three Health Equity Domains Only.

**Additional file 2:** Qualitative Codebook of the Three Health Equity Domains from Health Equity Implementation Framework.

## Data Availability

Data sharing is not applicable to this article as no datasets were generated or analyzed during the current study.

## Abbreviations

EPIS: Exploration, Preparation, Implementation, Sustainment
i-PARIHS: Integrated Promoting Action on Research in Implementation in Health Services
U.S.: United States
