# Supplementary material for: A more practical guide to incorporating health equity domains in implementation determinant frameworks
Source: Implement Sci Commun. 2021 Jun 5;2:61. doi: 10.1186/s43058-021-00146-5 (PMC8178842; doi:10.1186/s43058-021-00146-5)
Supplement: Supplementary file 1 — Additional file 1: Health Equity Implementation Framework Interview Guide: Three Health Equity Domains Only. [file 43058_2021_146_MOESM1_ESM.docx]

Interview Guide Questions for Three Health Equity Domains

Introduction

This is a semi-structured interview guide, from which the interviewer selects questions asked of participants. Only the three health equity domains are listed here. Our team has used and piloted many of these questions and retained them in our interviews. You will need to also align interview guide questions according to an established implementation determinant framework. When developing an interview guide, in addition to aligning it and informing it with these three health equity domains (and implementation science determinant from a selected framework), also consult with different experts in your clinical area or population to ensure you do not miss important concepts.

You will likely not be able to ask all these feasibly in one interview; select ones most relevant based on prior research and current gaps in knowledge. Questions are currently worded to be used in a formative evaluation (initial implementation diagnostic assessment) prior to implementation or to assess barriers / obstacles and facilitators / strengths to implementing an innovation equitably. You can edit them to assess implementation barriers / facilitators at a later time point during implementation. These questions will need to be reworded depending on participants interviewed -- e.g., patients, providers, leadership. You may need more than one question to assess each subdomain.

1. **Clinical Encounter (Patient-Provider Interaction)**

Have you ever had problems when you met with a doctor or nurse to talk about [innovation]?

Have you ever experienced problems when you met with a doctor or nurse at [healthcare facility]?

How do your conversations about [health problem or innovation] usually go with your provider?

Tell me about how your most difficult visits related to [innovation] usually go.

When you meet with patients from [marginalized group], how do you change or tailor the visit?

1. **Cultural factors of recipients**

**Provider and staff culturally relevant factors**

Demographics (e.g., neighborhood immigrant status) –

[recommend using standard questionnaire for this, only use interview questions to explore something pertinent to the implementation issue after reviewing questionnaire]

Unconscious / implicit bias –

[when interviewing providers and staff, although more likely to generate clearer and more accurate understanding from validated measures, some interview questions may be helpful]:

Tell me about the types of patients you see for [health problem] or [innovation].

What are some common challenges you run into when working with those patients?

[questions for patients about sociopolitical forces may also generate data on perceived provider bias]

Knowledge and attitudes –

What do you think about [innovation]?

What do you think about your patients who are [marginalized group]?

What are your thoughts on [health problem] at this time?

As a patient, do you think providers know how to help you with [health problem] or [innovation]?

Skillsets –

What has been your prior experience with [innovation] or treating [health problem]?

Do you feel you need additional training to [provide innovation]?

**Patient culturally relevant factors**

Medical mistrust –

What is your trust in [healthcare facility]?

Based on history in the past, do you believe providers at [healthcare facility] or for [health problem] will try to treat your problem the best possible way? Tell me about that.

How do you feel about your providers at [healthcare facility]?

How do you feel about going to get health care for [health problem], given your past experiences?

How do your friends, family, or peers talk about [healthcare facility]? What do you think about their views?

Health literacy and numeracy –

[consider following single question validated to detect inadequate literacy issues]

“How confident are you filling out forms by yourself?” [response: Likert scale from 0 to 4, all of the time, most of the time, some of the time, a little of the time or none of the time)

Do you have a hard time with medical statistics when you try to read them or your doctor talks to you about them? Tell me about that.

Demographics (e.g., neighborhood, immigrant status) –

[recommend generating this information from surveys, but interview questions can be used to clarify or expand as relevant]

Socioeconomic status (e.g., household income, net wealth, health insurance status, education level) –

[recommend generating this information from surveys, but interview questions can be used to clarify or expand as relevant]

Expectations about therapeutic relationships –

As a patient, what do you expect of your provider when you discuss [health problem]? What do you want your provider to know?

How do you want your provider to interact with you?

Biology/genetics –

As a provider, are there any other conditions that are contraindicated for treatment using [innovation]?

As a patient, do you have any other health issues that make you not want to [use innovation]?

Do your other health problems make [using innovation] harder? Easier?

1. **Societal Context**

**Economies**

How do you and most people you’re around have income or pay for things?

Are you able to get the health care you need, financially?

Do you have insurance?

As a provider, do you take insurance? Which ones?

How does [innovation] get paid for at your [healthcare facility]?

How does this affect who receives [innovation]?

**Physical Structures**

Location of patients to healthcare facilities –

How difficult is it for patients to get to [healthcare facility]?

State of healthcare facilities –

When you visit [healthcare facility], what do you expect to see?

What would you like to see, as far as the physical amenities and layout?

What is the state of the physical buildings of [healthcare system]?

Does that affect your care?

Transportation needs –

What transportation needs to be arranged to receive [innovation] at [healthcare system]?

Is it safe?

Is it reliable?

Language spoken or on materials –

Are you able to receive information and doctor’s advice in your preferred language?

If it is translated, is it good quality?

How do you handle it when you see patients of [marginalized group] and they do not speak your language?

Available structures in one's living environment to use innovation (e.g., senior center, safe walking paths) –

When your patients leave the hospital, do they have what they need in their environment to [use innovation]?

As a patient, when the doctor tells you to [use innovation], are you able to where you live or work?

Local businesses –

What local business, like [example] make it easier for you to [use innovation]?

Is there a lack of these business that makes it harder to use innovation?

**Sociopolitical forces**

Policies – Which government / hospital policies do you know of that affect implementation of [innovation]?

Political support – Are there any political issues currently affecting [innovation / health problem]?

Laws – Are there any laws or legal issues you can think of that might make it harder or less likely for people to [receive innovation]?

Local cultural movements (e.g., media campaigns) – Has anything been going on in your community about [innovation / health problem]? What do you think about that?

Do you feel there is a stigma against [marginalized group / health problem / innovation]? If so, tell me more about that.

Broader social movements (e.g., Black Lives Matter, Deplorables) –

Are there any things happening in society right now that affect your health care for [health problem] or your ability to provide care using [innovation]?

Recently, there has been more attention to [broader social movement]. How might this affect your health?

How might it affect your ability to [receive innovation]?

Structural discrimination (e.g., racism, ableism, classism, heterosexism, transphobia) – In the survey / screening, you stated you were [marginalized group, e.g., Black, queer] or [reported you’ve had experiences with discrimination before]. How do you feel that relates to your experience with [health problem]?

How do you feel that relates to getting care at [healthcare system]?

How do you feel that affects you [starting / receiving innovation]?

Have you ever felt treated differently in getting treatment for [health problem]?

Do you feel not understood by your doctor or nurse?

Have doctors/ nurses seemed like they were “keeping their distance” from you because you had [health problem]?

**Final question**

Knowing we are interested in [innovation] for [marginalized group], is there anything else you’d like to add?
